# Spatiotemporal behavior pattern differentiation and preference identification of tourists from the perspective of ecotourism destination based on the tourism digital footprint data

**DOI:** 10.1371/journal.pone.0285192

**Published:** 2023-04-28

**Authors:** Wei Dong, Qi Kang, Guangkui Wang, Bin Zhang, Ping Liu

**Affiliations:** 1 Department of Environmental Design, School of Art and Media, Xi’an Technological University, Xi’an, China; 2 Department of Planning and Construction Research Institute, Northwest Branch of Beijing Tsinghua Tongheng Urban Planning & Design Institute, Beijing, China; 3 Department of Product Design, School of Art and Media, Xi’an Technological University, Xi’an, China; University of the Chinese Academy of Sciences, CHINA

## Abstract

Tourist impact management in ecotourism destinations requires an accurate description of tourists’ spatiotemporal behavior patterns and recreation preferences to minimize the ecological environmental impact and maximize the recreation experience. This study classified tourist behaviors into five typical behavior patterns based on the digital footprints of tourists visiting Ziwuyu of the Qinling Mountains, including 348 traveling tracks and 750 corresponding geotagged photographs: short-distance, traversing, reentrant, large loop, and small loop. Furthermore, each behavior pattern’s recreation preference was identified using photograph analysis. Tourists with large-loop and reentrant behavior patterns have 89.8% and 30% chances of visiting Jianshanding, respectively. Key protected areas are faced with the risk of ecological environmental damage. Based on the behavior pattern differentiation and preference of tourists, this paper provides a decision-making basis for the classified management and guidance of tourists in ecotourism destinations. It has reference value for the management of similar ecotourism destinations.

## Introduction

In the premise of “protecting natural resources of national parks, ecotourism destinations are committed to provide high-quality ecotourism services for tourists [1]. The key is to achieve a balance between human recreational activities and nature conservation [2]. Global continental ecotourism destinations witness approximately 8 billion tourist arrivals each year [3] (estimated in 2015), with an economic output of 6 trillion dollars [4] (estimated in 2019). Tourism activities in ecotourism destinations that are designed with the environment as the main attraction play an important role. However, frequent recreational activities have invariably had a negative impact on the ecological environment, such as plant trampling, a decrease in soil organic substances, the destruction of aquatic ecosystems, and the loss of wildlife habitat [5, 6]. Therefore, effective tourist management is crucial for avoiding ecological risks, improving the recreation experience, and achieving the sustainable development of ecotourism [7].

Tourist impact management effectively solves the conflicting aims of tourism and environmental protection in ecotourism destinations [8]. The objective of tourist impact management is to achieve a dynamic balance between minimizing impact on resources and the environment and maximizing recreation experience quality at ecotourism destinations [9]. Limits of acceptable change (LAC), recreational opportunity spectrum (ROS), visitor activities management planning (VAMP), tourism optimization management model (TOMM), visitor experience and resource protection (VERP), visitor use management (VIM), and other modes of tourist impact management are currently in use [8]. Except for differences in specific operations, they have the same core idea: paying attention to the recreation experience quality of tourists and emphasizing the control of tourist impact while meeting the expectations of tourists. More flexible management measures shall be implemented according to the recreational process and characteristics of tourists [1, 9]. Under the guidance of the aforementioned theories, studies have been conducted on tourists’ spatiotemporal behaviors with the goal of tourist impact management. These studies aimed to help identify tour routes, tour locations, and usage levels in key protected areas to provide “early warnings” of ecological risks in ecotourism destinations to minimize the impact of tourism activities on the ecological environment [10].

Most studies on tourist spatiotemporal behavior patterns in ecotourism destinations have mainly focused on the general description of the level of recreational use [11] but lack an in-depth analysis of the spatiotemporal behavior patterns of tourists and the characteristics of nonspatial tourists [12]. In particular, few studies have been conducted on tourist behavior patterns and their recreation preferences from the perspective of tourist impact management [13]. Stamberger L et al. demonstrated in a study on Denali National Park and Preserve that tourists exhibit different behavior patterns in recreational activities at ecotourism destinations, and identifying areas that may suffer environmental degradation under different patterns is critical to ensuring the effectiveness of tourist impact management [14]. Furthermore, a recent study by Väisänen T et al. found significant differences in recreation preferences among different tourist groups using geotagged photographs, emphasizing the importance of understanding tourist preferences in ecotourism destinations [15]. Given the above, it is unscientific and usually ineffective to guide tourist impact management with the research results not considering tourists’s behavior patterns and recreation preferences, which has been agreed by scholars such as Beeco JA et al. [1], Sisneros-Kidd AM et al. [16], and Wilkins EJ et al. [17].

The research objectives of this study are listed as follows: (1) guided by tourist impact management and based on the spatiotemporal factors such as visiting duration, route length, and visits to scenic spots, the typical spatiotemporal behavior patterns of tourists in ecotourism destinations were identified. The negative impacts of each pattern on key ecological reserves were evaluated. (2) Tourist recreation preferences were investigated under various patterns. Based on this, the recreation experience of tourists in each pattern was specifically optimized. Furthermore, alternative schemes for tourists entering key ecological reserves based on their recreation preferences were established, and guidance was provided through design strategies to achieve more scientific, effective, and humanized tourist impact management.

This paper is organized as follows: in the first section, the research progress of current tourist spatiotemporal behaviors is summarized from the aspects of data, methods, and applications. In the second section, the current situation of ecotourism in Ziwuyu, which is the research object, the acquisition and processing of tourists’ digital footprints, and the methods used in this study for identifying tourists’ behavior patterns and recreation preferences are discussed. The empirical research is presented in the third section, focusing on the differentiation of spatiotemporal behavior patterns of tourists in Ziwuyu; additionally, the differences in recreation preferences among different behavior patterns are detailed. This study’s summary, recommendations, discussion, and prospects are presented in the final section.

## Research progress of tourists’ spatiotemporal behaviors

### Digital footprints enabling the accurate description of tourists’ spatiotemporal behaviors

After nearly half a century of development, the time-geography-centered research on spatiotemporal behaviors has become an important perspective for understanding the correlation between geographical space and human behaviors [18]. The development of surveys on spatiotemporal behaviors can be divided into four stages. The first stage mainly involved on-field behavioral observations and interviews to acquire information on the general behavior of tourists through careful observations. However, interviews are time-consuming, laborious, and limited in sample size and thus pose the risk of over-interpretation even though they can provide a deeper understanding [19]. The second stage involved using activity logs data to summarize the general spatiotemporal patterns of tourists. Mings RC, the representative scholar, summarized four typical behavior patterns through activity routes based on the activity logs of 600 tourists [20]. However, due to the over-reliance on memories and cognitive level of the respondents, surveys based on activity logs yield poor accuracy in the aspects of routes, behavior chain, and duration of stay [21, 22].

With the development of GPS technology, positioning navigation, and timing function enabled high-accuracy and continuous information of location, speed, and time, enabling the accurate acquisition of the spatial position and state of tourists. The survey of tourists’ spatiotemporal behavior henceforth entered the third stage of development. Using GPS track data, Beeco JA et al. identified the potential recreation conflict areas of different tourist groups in urban parks [23]. Korpilo S et al. also used GPS track data to study the impact of the environment on tourist’ tour routes in complex road networks [24]. Taking ski tourism in Tatra National Park as an example, Bielański M et al. systematically explained how to use GPS track data to monitor tourist activities’ location, intensity, and duration in fragile environments [25]. Based on the GPS track data and questionnaire information of tourists in Hong Kong Ocean Park, Huang X et al. accurately described three types of spatiotemporal behavior patterns of tourists visiting Hong Kong [26]. The track data obtained using portable GPS devices have been widely used in tourist spatiotemporal behavior studies.

However, the self-collection of GPS track data is time-consuming and labor-intensive, and samples are usually limited to a specific season. Furthermore, tourists are more likely to change their routes consciously if they are aware that their spatiotemporal behaviors are being monitored. Tourists can leave electronic footprints along with positioning information on the network while traveling in the information age. Such digital footprints can paint a complex picture of tourists’ individual and group behaviors [27]. The fourth stage is the research on tourists’ spatiotemporal behaviors based on their digital footprints. American scholar Girardin F proposed the concept of digital footprint [28]. Compared with traditional sociological survey methods such as spatiotemporal diaries, brain cognitive maps, observational methods, questionnaires, and interviews, the digital footprint can record and describe the spatiotemporal behaviors of tourists more accurately. Compared with GPS location trackers, digital footprint requires less effort, can yield a larger sample size, and covers the four seasons. Thus, this data source has a better representation of tourists’ spatiotemporal behaviors.

In microscale studies on digital footprints, Wood SA et al. estimated the visiting behaviors of tourists at 836 scenic spots worldwide through the geo-referenced photographs and photographers’ information from Flickr, an online social media site. They compared the results with the experience data for these scenic spots. They found that digital footprint can be a reliable representation of empirical visitation rates [29]. Väisänen T et al. analyzed photographs captured by tourists visiting Finnish national parks using computer vision methods such as semantic clustering, scene classification, and object detection. They identified the recreation preferences of different tourist groups based on the photographs in digital footprints [15]. Liu Y obtained the jogging track data of citizens in urban parks from social media to analyze the spatiotemporal behavior patterns of jogging in urban parks and demonstrated through regression analysis that designated jogging tracks and aquatic installations in parks have a positive impact on jogging [30]. The preceding studies show that geographic reference and track data can be used to quantify tourist behavior characteristics across multiple dimensions. Such digital footprints are distinguished by their timeliness, universality, and authenticity, and they play an indispensable role in the study of tourists’ spatiotemporal behaviors. They are also becoming an important means of accurately describing tourists’ behaviors. However, few studies have successfully combined photograph and track data. Therefore, solving the problem of integrating multisource data in empirical research is critical.

### Synchronous development of studies on behavior patterns and recreation preferences

The research on tourists’ spatiotemporal behaviors exhibits a trend of synchronous development between studies on behavior patterns and recreation preferences. In terms of behavior patterns, based on the spatial information of stay and the sequence information of visited places, Orellana D et al. proposed two methods for aggregating tourist behavior patterns: movement suspension patterns (MSPs) and generalized sequential patterns (GSPs) [31]. By introducing the research methods of time-geography into the study on tourist behaviors, Huang X et al. verified the feasibility of using time, spatial, and activity area information as the clustering elements of tourists’ spatiotemporal behavior patterns [26]. Kidd AM et al. extracted the start and end time of travel, place, and duration of stay, and other information from GPS tracks of tourists’ vehicles for cluster analysis and divided the behavior patterns of tourists visiting the Moose-Wilson corridor of Grand Teton National Park into three types: opportunistic commuters, wildlife/scenery viewers, and hikers [13].

With respect to the study on recreation preferences, the differences in the spatiotemporal distribution of tourists with different properties in scenic spots have been studied extensively. Taking Sarawak Malaysia Total Protected Area as an example, Abdurahman AZ et al. conducted spatial and temporal analyses of the monthly average trend of local and foreign tourists. They identified the months and places witnessing the maximum visits [32]. Huang Q et al. quantized the spatiotemporal distribution characteristics of recreational behaviors of backpackers with different travel modes in Beijing [33]. Furthermore, environmental factors influencing recreation preferences have been investigated to establish a link between tourist behavior and tourist destinations. For example, Van Vliet E et al. conducted an online selection experiment of virtual parks to investigate the impact of park environmental factors on tourists’ recreation preferences. They discovered that natural factors such as trees and flowers significantly impact tourists’ recreation preferences [34]. Veitch J et al. studied the environmental factors affecting the participation of older people in physical exercise and social activities in parks. They found that tree-shaded areas and quiet trails are more important to them [35].

The research on tourist behavior patterns has mainly focused on the similarity of tourist spatiotemporal behaviors. Based on this, structured classification has been conducted to improve scenic area management. The study of tourists’ recreation preferences focuses on the differences in the tourists’ spatiotemporal behaviors and the optimization of tourists’ recreation experiences by identifying different preferences. However, the common purpose of these two types of studies is to describe tourists’ behaviors in scenic area. The current study investigated the dilemma of tourist management in ecotourism destinations in combination with these two modes of thinking.

### Considering the subject and object in the research on tourist spatiotemporal behaviors

The application of tourists’ spatiotemporal behavior research can be summarized into two categories: (1) focusing on the recreation experience optimization of the recreation subject and (2) focusing on scenic area management. In terms of the recreation experience optimization, Huang X et al. proposed that tourist destinations should control the tourist flow according to different patterns, reduce waiting time at venues and facilities, and provide targeted services to improve the quality of the tourist experience. To this end, they studied the spatiotemporal behavior pattern of tourists in Hong Kong Ocean Park [26]. Taking tourist groups as the research object and through the quantitative analysis of their spatiotemporal behavior characteristics, Zhao X et al. proposed measures such as improving the travel route design of the group and adding small commercial spots to improve the travel experience of tourist groups [36]. Xia JC et al. considered tourists visiting Phillip Island, Victoria, Australia, as an example, and divided the tourism market segments by identifying several important mobility patterns. This helped park managers decide when to open scenic spots and how to arrange the daily activities at scenic spots to meet the needs of tourists and improve their experience [37].

Stamberger L et al. determined the areas that might suffer from environmental degradation by studying the spatiotemporal behavior patterns of tourists in remote areas for the research on tourists’ spatiotemporal behaviors to support scenic area management, using Denali National Park and Preserve as an example. They demonstrated the practical significance of studying tourists’ spatiotemporal behaviors in ecotourism destinations for tourist impact management [14]. To minimize the impact of tourists on the ecological environment, Kidd AM et al. analyzed the spatial behavior patterns of tourists traveling by bus in Grand Teton National Park and analyzed the classified benchmark management of tourists in ecotourism destinations [13]. D’Antonio A et al. used GPS tracking to study the spatial behavior of tourists in three parks. It was reported that understanding the spatiotemporal behavior characteristics of tourists is of great significance for protecting ecological environments such as national parks and improving the tourist experience [38].

From the above discussions, it is obvious that the application of tourist behavior research presents a binary differentiation. Some researchers focused on studying tourist behavior to improve the recreation experience, whereas some aimed to guide the tourist management of national parks by analyzing tourists’ spatiotemporal behaviors. However, according to tourist impact management theory, good coordination between environmental protection and recreation experience is required for ecotourism destinations. Thus, this study aimed to reduce the impact of ecotourism destinations on the environment and resources while increasing the quality of the recreation experience by revealing the differentiation of tourists’ spatiotemporal behavior patterns and preferences. Additionally, Ziwuyu in the Qinling Mountains was chosen to conduct empirical research.

## Behavior research design under tourist impact management

### Current tourist impact management of wuziyu, qinling mountains

The Qinling Mountains, together with the Alps in Europe and the Rocky Mountains in North America, are crowned as the “three mountains” of the world. The Qinling Mountains act as the dividing line between the north and south climate in China, constitute an important ecological safety barrier and water conservation area, and is one of the regions with the richest biodiversity in the world. The Shaanxi section of the Qinling Mountains has an average annual CO2 absorption of 146.7 million tons (statistical data collected in 2021) [39], which is significant in coping with global climate change. The Qinling Mountains are characterized by uniqueness, complexity, and ecological sensitivity. With the continuous increase in the regional population, resource development, and urbanization, the Qinling Mountains are facing increasingly severe environmental stress, and the contradiction between social development and ecological environment protection is becoming increasingly prominent. The Qinling Mountains are connected with the plains through 72 valleys. The valley’s mouth-shaped area is endowed with unique natural characteristics, rich culture and historical resources, and a complete ecosystem. As a hub connecting protected and nonprotected areas, it is also the area where the conflict between development and protection is most prominent.

Ziwuyu is an important area with heritage sites, recreational resources, and ecological buffer zones located in the transitional zone of the mountain and plain ecosystems. Ziwuyu was chosen as the research object in this study to investigate the tourist impact management of ecotourism destinations. On the one hand, Ziwuyu, a typical ecotourism destination popular with tourists, can meet various recreational needs such as being close to nature, cultural education, mountaineering expeditions, and local experiences. Ziwuyu, one of the 72 valleys of the Qinling Mountains and the birthplace of Taoism, is located in Chang’an District, Xi’an City, with 108.89 degrees east longitude and 34.03 degrees north latitude, a length of 7.44 kilometers, and an area of 17.53 km^2^. Ziwuyu is an important cultural heritage-bearing area, recreational resource gathering area, and ecological buffer zone located at the ecotone of the mountain and plain ecosystems. Therefore, conducting research on tourist impact management in ecotourism destinations with Ziwuyu as the object is appropriate. On the one hand, as a typical ecotourism destination popular with tourists, Ziwuyu has numerous cultural and historical relics, such as Jinxian Temple, Cliff Stone Carvings, and Xuandu Temple, as well as over 200 types of precious medicinal materials such as Poria cocos, Gastrodia elata, and Ganoderma lucidum. Ziwuyu, with its lush peaks, beautiful scenery, dangerous terrain, and ancient trees, can accommodate a wide range of recreational needs, including getting close to nature, cultural education, mountaineering exploration, and local experience. Ziwu Valley is 29.70 km from downtown and can be reached in one hour by car. It is the valley in the Qingling Mountains closest to downtown. Previously, it could receive 80,000 to 100,000 tourists on the weekend during peak season. n the other hand, Ziwuyu is faced with urgent ecological protection. In 2019, Xi’an Qinling Mountains Ecological and Environmental Protection Regulations (“Regulations”) were issued, and the Qinling Mountains Ecological Protection Station was set up in Ziwuyu, limiting the daily count of tourists entering the valley to no more than 3000. In addition, numerous rigid control measures were implemented. As an ecotourism destination, the contradiction between recreational activities and ecological protection in Ziwuyu has become prominent.

Ziwuyu receives tourists with different tour motivations, recreational behaviors, and local villagers. Thus, taking Ziwuyu as a research object, it is typical to study the differentiation of tourist behavior patterns and their recognition of recreational references from the perspective of tourist impact management in ecotourism destinations. The terrain in Ziwuyu is higher in the south than in the north, and the valley mouth is on the northernmost side, at an elevation of about 500 m. Jianshanding, located in the valley’s southeast corner, has an elevation of 1584 m and is a protected area as defined by the regulations. According to the requirements, measures should be implemented to prevent the entry of people who are unrelated to environmental protection. In Ziwu Valley, the terrain is high in the south and low in the north. Yukou is located on the valley’s northernmost ridge at an elevation of about 500 m. According to the local tourist impact management policy, the elevation of the peak at the southeast corner of the valley reaches 1584 m. It thus belongs to the key protection area specified in the Regulations, which primarily performs the ecological protection function. Closed measures should be implemented to limit human activities to the greatest extent possible and to prohibit the entry of personnel unrelated to environmental protection, following the requirements. Such rigid constraints can help achieve tourist impact management but will also inevitably diminish the recreation experience of tourists and villagers. However, the control of the tourist count cannot eliminate the risk of environmental damage due to the ignorance of the differentiation of tourists’ spatiotemporal behavior patterns. Therefore, this study developed a method to identify behavior differentiation and preferences to support more flexible and effective tourist management in ecotourism destinations.

### Acquisition and processing of tourist digital footprints

Tourism digital footprint includes GPS tracks, location photographs, travel logs, and online reviews, with a collection time span of several years. It offers the advantages of accurate positioning, rich behavior information, low cost, and a large sample size [40], enabling better control of the heterogeneity of individual recreational behavior [41]. Its effectiveness in the study of tourists’ spatiotemporal behaviors has been verified [42]. However, the digital footprint has certain shortcomings when applied to behavioral research, such as changes in data accessibility, exclusion of non-user individuals, and insufficient user attributes [43]. Thus, its reliability should be carefully considered when using it for research [44]. These are not insurmountable obstacles. The integration of data from different platforms can help increase the representativeness of samples and enable better evaluation of the heterogeneity preference of tourists in ecotourism destinations [45]. In addition, appropriate data cleaning can improve the robustness of the study [10].

**Fig 1:**
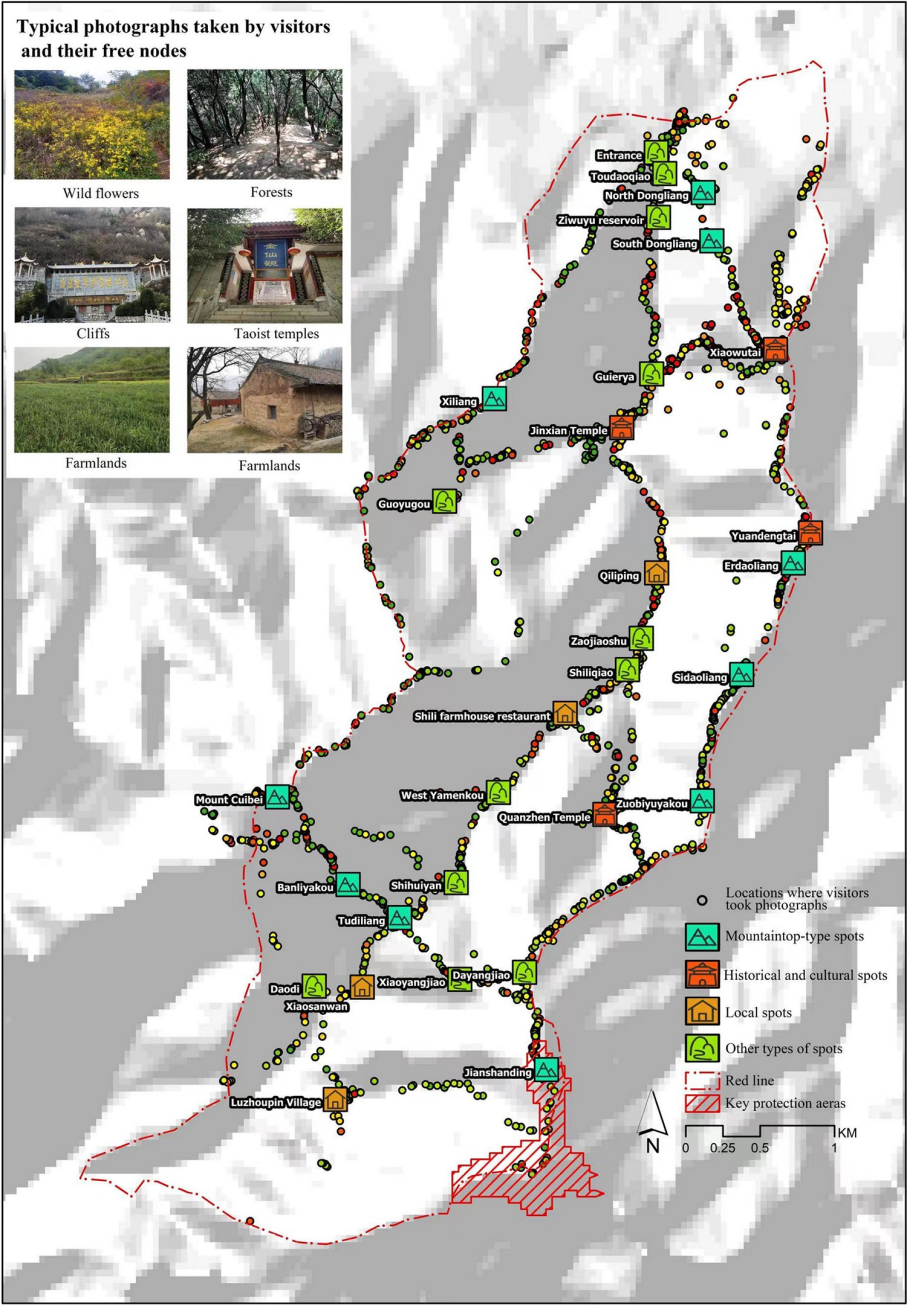
Geotagged photographs and codes. Reprinted from [https://www.usgs.gov/centers/eros/data] under a CC BY license, with permission from [United States Geological Survey], original copyright [2000]. Reprinted from [https://www.2bulu.com/] under a CC BY license, with permission from [Shenzhen Bubulu Information Technology Co., Ltd.], original copyright [2020].

Taking the tourism digital footprints from http://www.foooooot.com/and https://www.2bulu.com/, the two popular Internet track-sharing platforms widely used by Chinese people, as the data source. According to the privacy policies of the two platforms, users have authorized the two platforms to collect information such as personal information, activity track, and photos, as well as to use anonymized and aggregated data. Yao Q et al. and Zhang H et al. demonstrated the reliability of the two data sources in the study of tourist behavior [46, 47]. With the support of Foooooot and 2bulu, we obtained the tourism digital footprint of Ziwuyu for March 2017-March 2020, including 482 anonymous tourist tracks and 2558 geotagged photographs (Fig 1). All tourists are anonymous because this is a retrospective study of archived samples. Before we could access the data, it was completely anonymized. The ethics committee of Xi’an Technological University waived the requirement for informed consent, and the committee approved the study. A preliminary cleaning was performed on the collected track data to improve the reliability of the data for subsequent analysis. We deleted the samples that did not pass through the study area, the samples whose tracks were incomplete, and the samples wherein the positioning points were vague, and the recreational route could not be identified accurately. Furthermore, samples with a tour duration of less than 30 min and a route distance of less than 1.5 km were removed. Following cleaning, 348 effective tracks with an effective rate of 73% and 321,208 data-positioning points were obtained for analysis. Each point had the following attributes: track number, serial number, track point longitude, and latitude, the distance between two points, and timestamp. On this, the photograph data of the track were also cleaned. After deleting the samples with very low resolution, blurry images, too complex content, and unclear subjects, a total of 1792 effective photographs were obtained for the subsequent identification of the recreation preferences of tourists in Ziwuyu.

## Behavioral research method based on digital footprint

### Identification of tourist behavior pattern

Touring duration and locations are the key and active variables causing environmental destruction in ecotourism destinations and are also the key attributes for identifying tourist behavior patterns. To identify the pattern differentiation, we first extracted the spatiotemporal information contained in digital footprints. A variety of behavior tracks of tourists visiting Ziwuyu were clustered into several typical behavior patterns according to their spatiotemporal attributes in the ecotourism destination. Because there were both categorical and continuous variables in the cluster elements, the TwoStep Cluster in SPSS25.0 software was used to structurally classify the recreational spatiotemporal behaviors of tourists visiting Ziwuyu. The specific steps were as follows:

Step 1: The collected track data of tourists visiting the study area were inputted into the ArcGIS software. The track was expressed as a group of continuous spatial positioning points attached with coordinates, time, elevation, and other information.

Step 2: According to theories about tourists’ spatiotemporal behaviors, the cluster elements were divided into temporal and spatial elements. The time tourists entered and exited the study area and the time spent in recreation were all considered temporal elements. The number of visits to 30 scenic spots, the number of scenic spots visited, and the length of the tour route were all spatial elements. All of these were considered to be the initial cluster elements. Some scenic spots affecting the cluster were removed according to the stops of tourists (<5%) and the significance of cluster (≥0.05).

Step 3: The distance of all records was checked, and a CF classification tree was constructed. The records in the same tree node were highly similar, and the records with low similarity generated new nodes. The distance measurement considered a log-likelihood estimate as the index for measurement. The distance *d*_(*j*,*s*)_ between category *i* and *j* was expressed by the degree to which the log-likelihood value decreased after the combination of the two:

$$d_{\left(j,s\right)}=\varepsilon_{j}+\varepsilon_{s}-\varepsilon_{\left(j,s\right)},$$ (1)

$$\varepsilon_{v}=-N_{v}(\sum_{k=1}^{K_{A}}\frac{1}{2}\ln(\sigma_{k}^{2}+\sigma_{vk}^{2})+\sum_{k=1}^{K_{B}}E_{vk}),$$ (2)

$$E_{vk}=-\sum_{l=1}^{L_{k}}\frac{N_{vkl}}{N_{v}}\mathrm{lg}\frac{N_{vkl}}{N_{v}},$$ (3)

Where *ε*_(*j*,*s*)_ is the distance when cluster *j* and *s* are combined, *K*_*A*_ is the number of continuous variables used, *K*_*B*_ is the number of categorical variables, *L*_*k*_ represents the categories of the *k*-th categorical variable, *N*_*v*_ is the records of category *v*, *N*_*vkl*_ is the number that the *k-*th categorical variable with a value of *l* in cluster *v*, $\sigma_{k}^{2}$ is the estimator of the variance of the *k*-th continuous variable, $\sigma_{vk}^{2}$ is the estimator of the variance of all sample values of the *k*-th continuous attribute in cluster *v*, and *v* refers to category *j* or *s*.

Step 4: The nodes were classified based on the CF classification tree. The optimal cluster number was determined based on the BIC value of the Bayesian information discrimination formula and the changes in the shortest distance between clusters. Five typical spatiotemporal behavior patterns of tourists in Ziwuyu were finally obtained.

### Identification of tourists’ recreation preferences

Travel photographs are an important part of tourists’ tourism activities. This study adopted photograph analysis to quantitatively analyze tourists’ recreation preferences [48]. A total of 1792 tourist photographs were classified into five groups based on cluster analysis results. The number of users in each group was not equal to the number of photographs uploaded by each user. Given the complexity of photograph content and the sensitivity of the research method, and to avoid the interference of active users on research results [49], random sampling was conducted to improve the reliability of research results. The approach was: based on the number of tourist samples of each typical behavior pattern. Given that some users had not uploaded photographs, 30 tourists were chosen randomly as the initial samples for each pattern. After that, five photographs were chosen at random from the photographs uploaded by each user for further analysis. If a user uploaded fewer than five photographs, the photographs were drawn at random from other users with the same behavior pattern based on the difference. Finally, 750 sample photographs were obtained to identify tourist recreation preferences under typical behavior patterns.

First, the 750 sample photographs were coded freely using the NVivo 10 software to interpret the content of the photographs. No more than four free nodes were selected for each photograph. Due to the various categories of free coding, the internal differences and connections among them were determined. In accordance with the content characteristics, the node categories were determined to complete the axis coding, and the differences among categories were analyzed. For instance, forests, shrubs, streams, and flowers belong to the category of the natural landscape. In contrast, temples, Taoist temples, door plaques, memorial gates, and scriptures belong to the category of historic culture. A total of four first-level codes were obtained: natural landscape, historic culture, native village, and mountaineering expedition. The frequency statistics of each free node and subtree node were exported using the statistical function of NVivo 10 to quantize tourists’ recreation preferences under different patterns. The reliability of the photograph codes was assessed after the completion of coding. In this study, 1667 nodes were coded in the first round, among which 34 nodes were modified and deleted during the second round of coding. A total of 1630 nodes were finally coded, with an agreement degree of 97%, indicating that the coding performed in this study yields high stability and reliability [50]. It is thus feasible to conduct further result analysis.

## Identification of tourist spatiotemporal behavior patterns and recreation preferences

### Overall characteristics of tourists’ spatiotemporal distribution

From the overview of the sample (Table 1), men accounted for 72%, of the tourists visiting Ziwuyu, much higher than that of women. The proportion of elderly, middle-aged, and young people was 76%, 16%, and 8%, respectively, indicating that elderly tourists are the main visitors to Ziwuyu. Regarding temporal distribution characteristics, 34% of tourists chose to travel in autumn, 26% in winter, 24% in spring, and 16% in summer. In terms of the tour duration, 55% of tourists toured for 4–8 h, and 34% of tourists visited for less than 4 h. Furthermore, 38 tourists visited for more than 8 h, accounting for 11% of the sample.

**Table 1:** Overview of the study sample.

		Sample size	%			Sample size	%
**Gender**	Men	178	72%	Route distance/km	<4	16	5%
	Women	69	28%		4–8	59	17%
**Age**	Young people	20	8%		8–16	207	59%
	Middle-aged	40	16%		>16	66	18%
	Elderly	187	76%	Average speed km/h	<2	146	42%
**Month**	Spring	84	24%		2–3	142	40%
	Summer	55	16%		3–4	44	13%
	Autumn	118	34%		>4	16	5%
	Winter	91	26%	Number of scenic spots visited	2–4	62	18%
**Tour duration/h**	<4	119	34%		5–7	103	30%
	4–8	191	55%		8–10	88	25%
	>8	38	11%		>10	95	27%

Note: In this study, the integrity of tourists’ GPS tracks was used as the standard to judge the validity of the sample. Some samples with unidentifiable age and gender information were also included in the study. Thus, in the statistical results in Table 1, the sum of the samples under the two attributes is not necessarily equal to the number of effective samples.

Concerning spatial distribution characteristics, 59% of tourists had a tour route of 8–16 km, 18% of tourists had a route of over 16 km, and 17% and 5% of the tourists had tour routes of 4–8 km and less than 4 km, respectively. Concerning the number of scenic spots visited, 30%, 25%, and 27% of tourists visited 5–7, 8–10, and more than ten scenic spots, respectively. Only 18% of tourists visited 2–4 scenic spots. Autumn was discovered to be the peak season for Ziwuyu, and the majority of tourists were elderly men; most of them chose a tour route of 8–16 km, with an average speed of less than 3 km/h, and visiting 5–7 nodes.

### Five typical spatiotemporal behavior patterns of tourists

According to the cluster analysis results (Table 2), the tourist behaviors of those visiting Ziwuyu were structured into five typical spatiotemporal behavior patterns with a proportion of 18.8%, 22.8%, 26.0%, 18.2%, and 14.2%, indicating a good cluster effect. The analysis of the clustering results from temporal and spatial characteristics revealed significant differences in the length of tour routes, number of scenic spots visited, and total number of tourist visits under the five patterns. The five spatiotemporal behavior patterns were named after the above information: short-distance, traversing, loop, large-loop, and small-loop. Each behavior pattern is explained in greater detail in the following text.

**Table 2:** Cluster analysis results.

Cluster elements	Cluster type				
	First	Second	Third	Fourth	Fifth
**Sample proportion**	18.8%	22.8%	26.0%	14.2%	18.2%
**Temporal elements**					
**Start time/hour: minutes**	10:37	10:45	10:24	8:51	9:33
**Leave time/hour: minutes**	14:06	14:40	16:21	16:23	15:48
**Actual duration of tour/hour: minutes**	3:29	3:55	5:57	7:32	6:15
**Spatial elements**					
**Entrance**	1	1	2	1	2
**Guierya**	1	1	2	1	2
**Jinxian Temple**	0	1	2	1	1
**Xiliang**	1	0	0	0	0
**Qiliping**	0	1	2	1	1
**Shili farmhouse restaurant**	0	1	2	1	0
**Banliyakou**	0	1	0	0	0
**Tudiliang**	0	1	1	1	0
**Xiaoyangjiao**	0	0	0	1	0
**Dayangjiao**	0	0	0	1	0
**Jianshanding**	0	0	0	1	0
**Quanzhen Temple**	0	0	0	0	1
**Zuobiyuyakou**	0	0	0	1	1
**Erdaoliang**	0	0	0	1	1
**Sidaoliang**	0	0	0	1	1
**Yuandengtai**	0	0	0	1	1
**Xiaowutai**	0	0	0	1	1
**North Dongliang**	0	0	0	0	0
**South Dongliang**	0	0	0	0	0
**Leave the study area from non-entrance**	1	1	0	0	0
**Route length/km**	5.8	10.1	12.8	16.8	13.7
**Number of scenic spots visited**	3	7	6	14	10

#### Pattern 1: Short-distance

**Fig 2:**
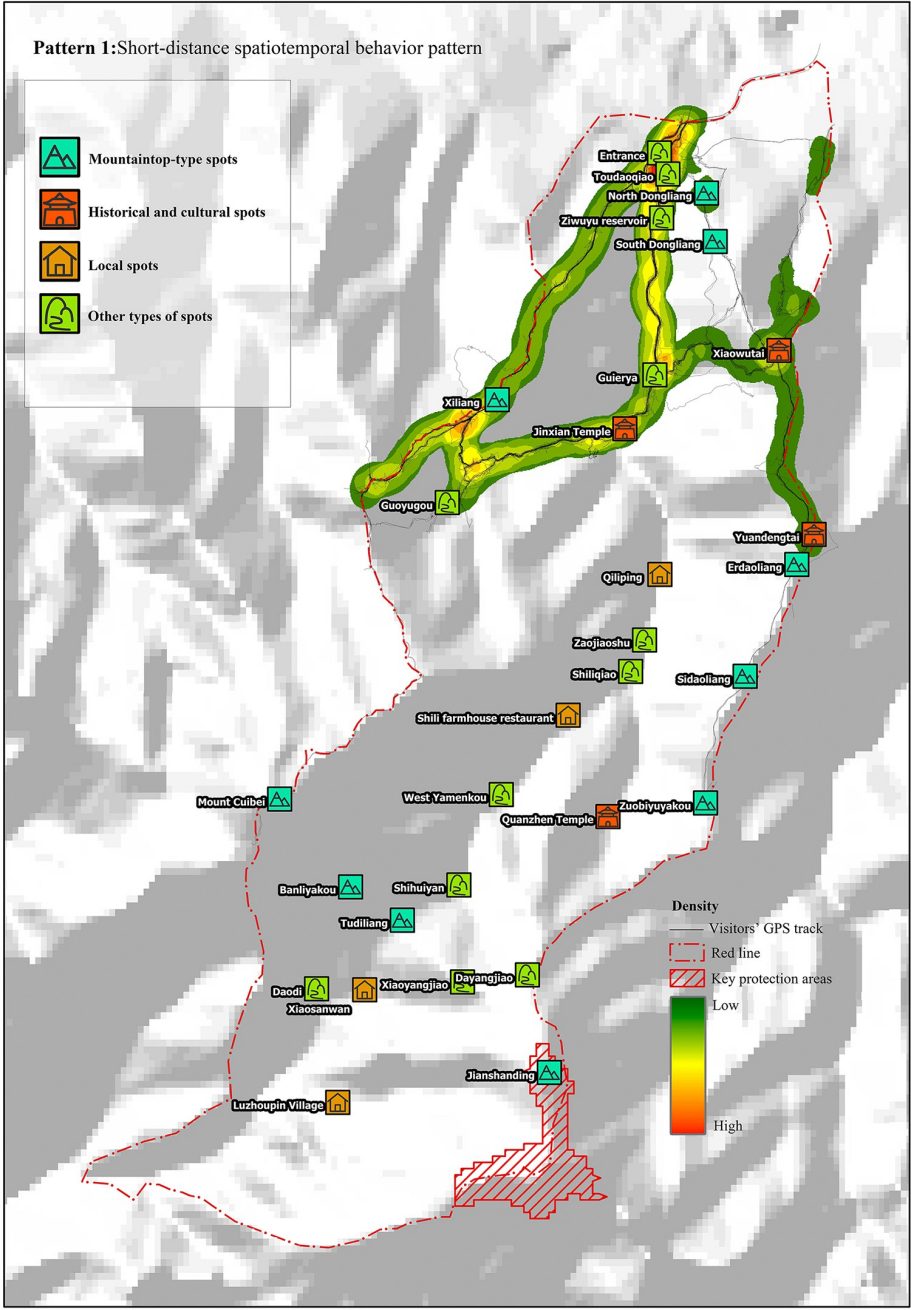
Short-distance spatiotemporal behavior pattern. Reprinted from [https://www.usgs.gov/centers/eros/data] under a CC BY license, with permission from [United States Geological Survey], original copyright [2000].

Tourists in this pattern had the shortest tour duration of approximately 3.5 h and the shortest route length of 5.8 km. As shown in the diagram of linear density analysis, such tourists were characterized by relatively dispersed tour routes and less scenic spots visited, mainly within 3 km from the entrance of the valley. The main nodes visited were the entrance, Guaierliang, and Xiliang, and the tourists left the study area from Xiliang (Fig 2).

#### Pattern 2: Traversing

**Fig 3:**
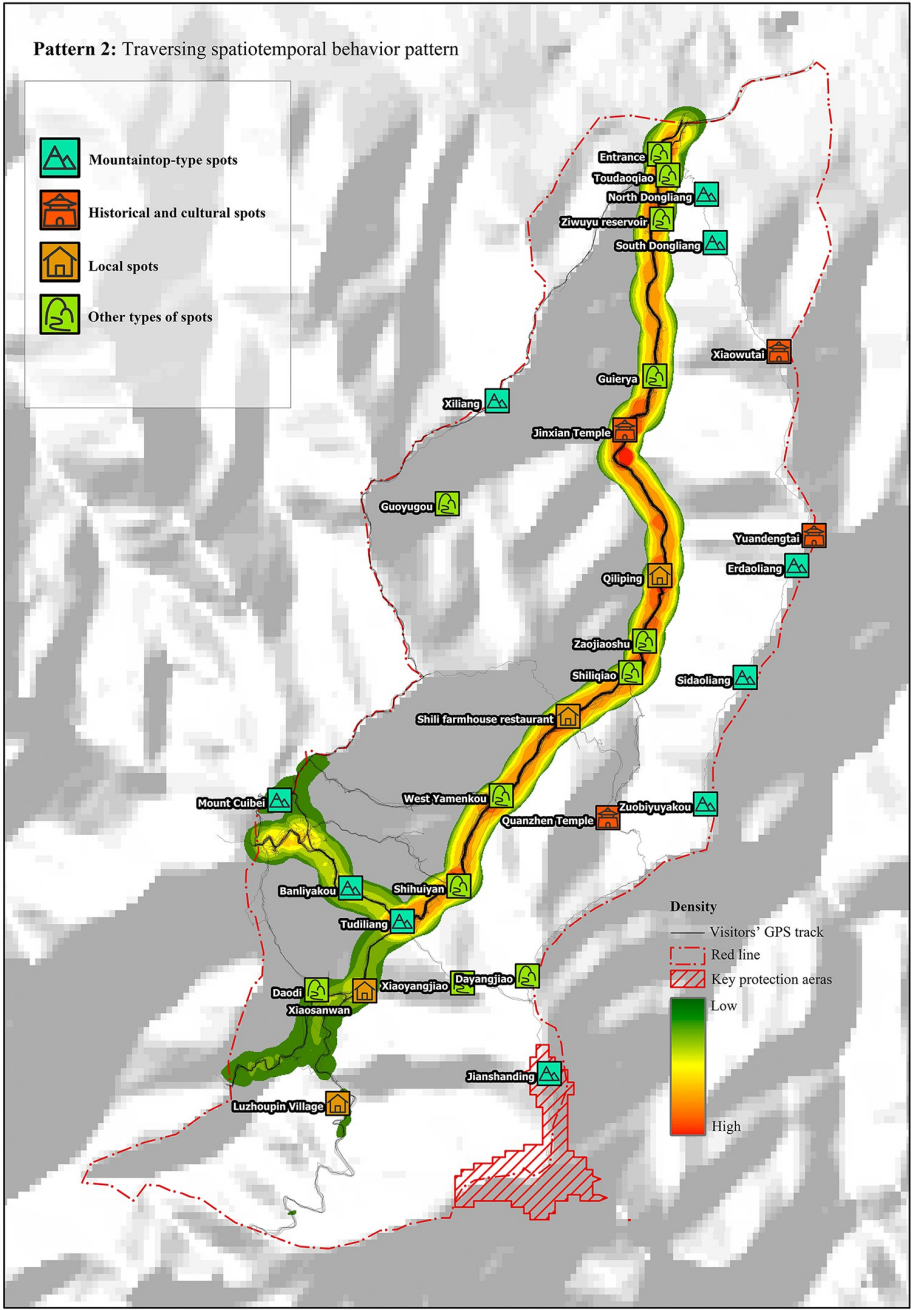
Traversing spatiotemporal behavior pattern. Reprinted from [https://www.usgs.gov/centers/eros/data] under a CC BY license, with permission from [United States Geological Survey], original copyright [2000].

Tourists in this pattern had a relatively short tour duration of approximately 4 h, but the route length was 10.1 km. According to the tour route (Fig 3), after entering from the entrance, tourists in this pattern passed through the nodes, including Guaierya, Jinxian Temple, Xiliang, and Qiliping, and then arrived at Tudiliang, from where the route started to disperse. They left the study area from the non-entrance.

#### Pattern 3: Reentrant

**Fig 4:**
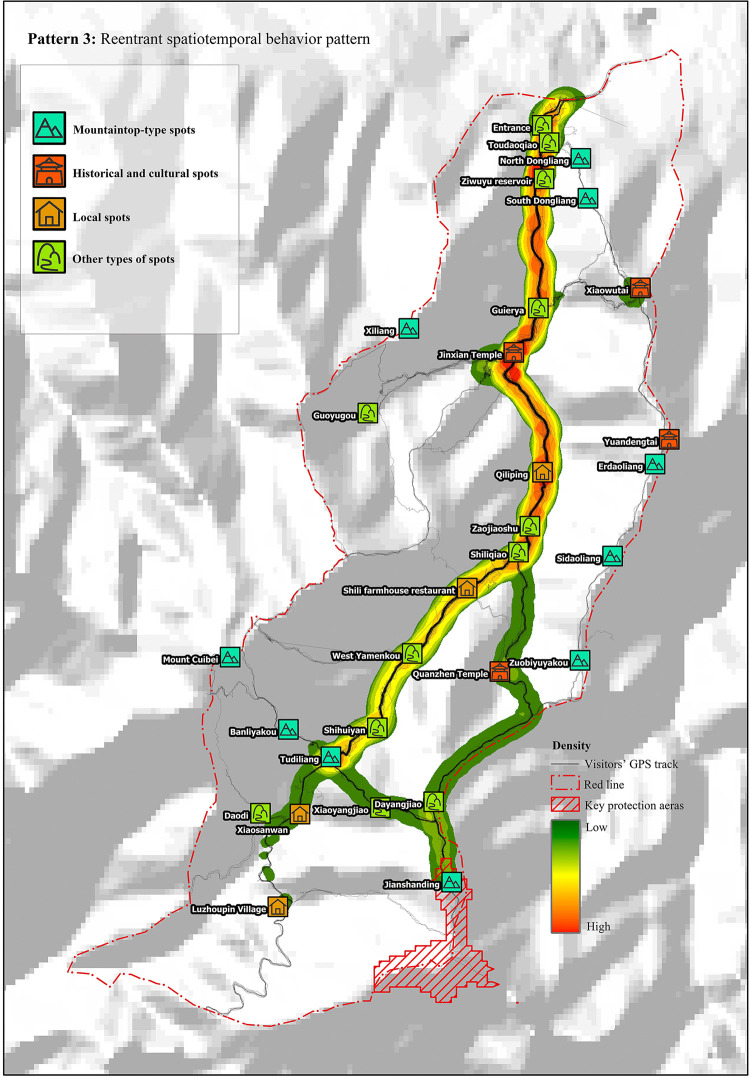
Reentrant spatiotemporal behavior pattern. Reprinted from [https://www.usgs.gov/centers/eros/data] under a CC BY license, with permission from [United States Geological Survey], original copyright [2000].

Tourists in this pattern visited for approximately 6 h. As shown in the diagram of linear density analysis (Fig 4), such tourists were characterized by relatively dispersed tour routes. The main route was to enter the mountain from the valley mouth and reach Tudiliang through Guaierya and Jinxian Temple. Due to physical constraints, it was impossible to complete the large loop; thus, tourists returned to the valley mouth using the same route or part of the same route.

#### Pattern 4: Large loop

**Fig 5:**
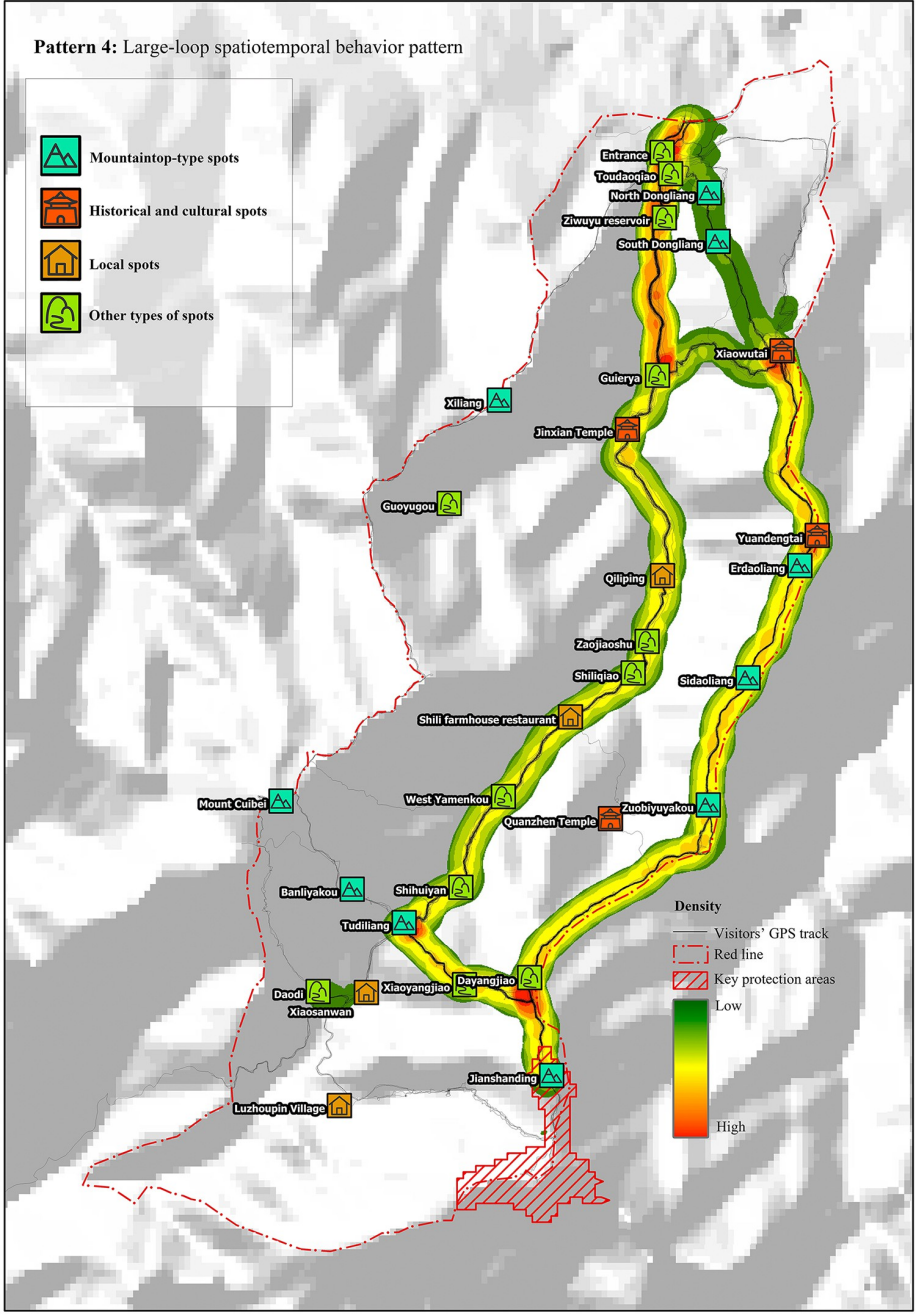
Large-loop spatiotemporal behavior pattern. Reprinted from [https://www.usgs.gov/centers/eros/data] under a CC BY license, with permission from [United States Geological Survey], original copyright [2000].

The duration of the tour was approximately 7.5 h, the longest tour route was approximately 17 km, and the number of scenic spots visited was up to 14. This pattern has high physical requirements for tourists. As shown in the diagram of linear density analysis (Fig 5), the main route of tourists in this pattern was to start from the entrance, pass through Guaierya, Jinxian Temple, Qiliping, Shili farmhouse restaurant, Yuandengtai, Dayangjiao and Xiaoyangjiao, Jianshanding, Zuobiyuyakou, Sidaoliang, Erdaoliang, Yuandengtai, and Xiaowutai, and finally scattering into two routes to go down the mountain.

#### Pattern 5: Small loop

**Fig 6:**
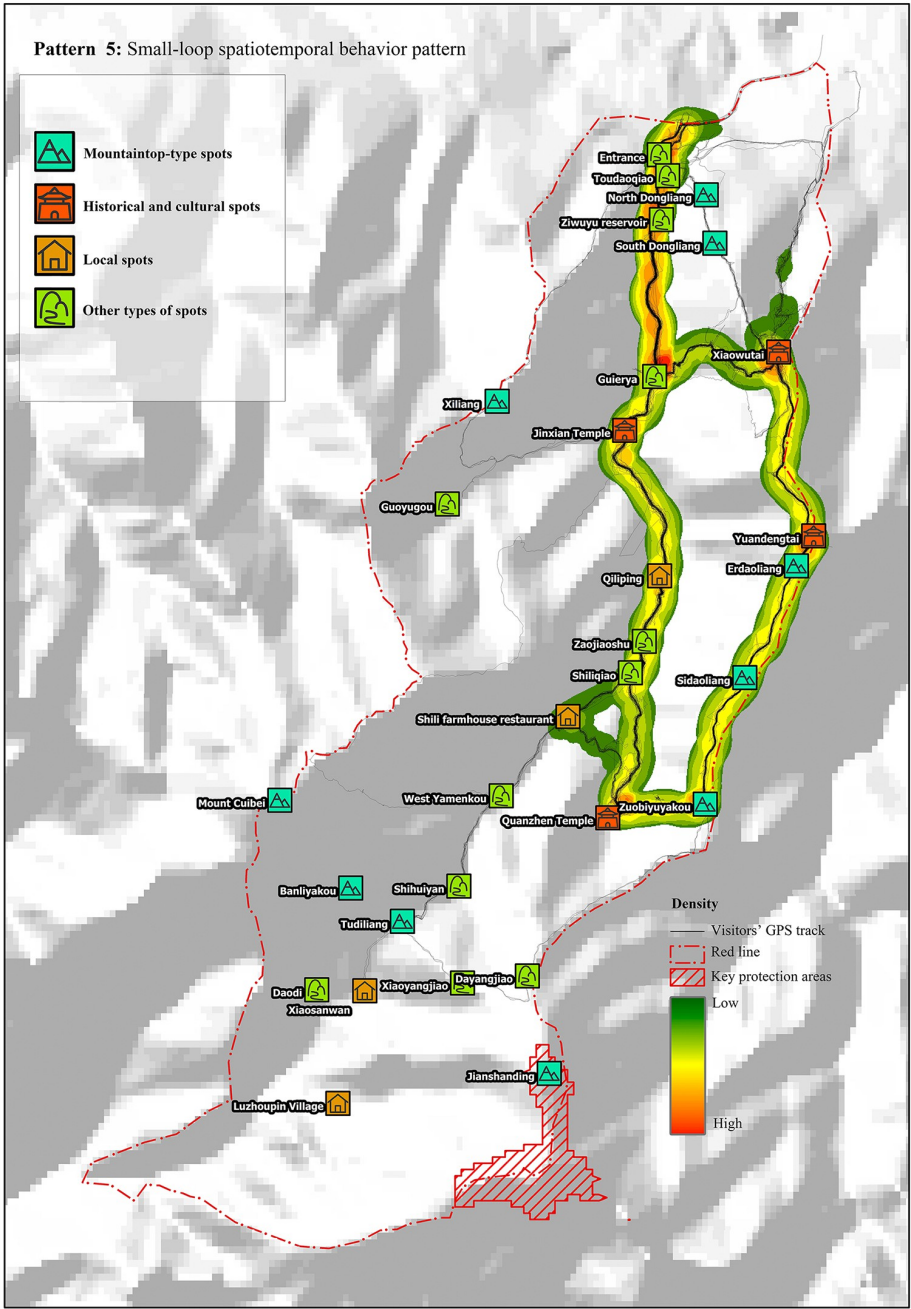
Small-loop spatiotemporal behavior pattern. Reprinted from [https://www.usgs.gov/centers/eros/data] under a CC BY license, with permission from [United States Geological Survey], original copyright [2000].

According to the cluster analysis results, tourists in this pattern accounted for 18.2% of the total tourists. Tourists in this pattern had a tour duration of 6 h and 15 min and a tour length of approximately 14 km, and the number of scenic spots visited was 10. The main route was to start from the entrance, pass through Guaierya, Jinxian Temple, Qiliping, Quanzhen Temple, Zuobiyuyakou, Sidaoliang, Erdaoliang, Yuandengtai, and Xiaowutai, and return to Guaierya to leave (Fig 6).

### Tourists’ recreation preferences and visit probability

To further understand tourists’ recreation preferences of different behavior patterns, further investigation was conducted using photograph analysis. The results (Table 3) revealed that forests, mountain roads, temples, and farmhouses, respectively, accounted for the largest shares in the four subtree nodes. According to the findings, tourists who are interested in natural landscapes are more likely to visit forests to take photographs. Tourists interested in mountaineering expeditions paid special attention to the rugged mountain roads. Temples and farmhouses drew the most attention from tourists interested in experiencing historic culture and native villages, respectively. Among the four first-level codes, natural landscape witnessed the maximum number of visits (57.55%), followed by mountaineering expeditions (25.46%) historic culture (11.60%), and native villages (5.40%). The statistical results revealed that tourists visiting Ziwuyu were mainly passionate about natural landscapes and mountaineering expeditions, supplemented by the experience of historic cultures and native villages.

**Table 3:** Node category and statistics of each behavior pattern.

Subtree nodes (%)	Free nodes	Number					Total (%)
		Pattern 1	Pattern 2	Pattern 3	Pattern 4	Pattern 5	
**(57.55%) Natural landscape (57.55%)**	Forests	112	107	104	107	91	521(31.96)
	Shrubs	66	30	37	57	36	226(13.87)
	Wild flowers	11	11	14	2	8	46(2.82)
	Grasses	6	2	14	12	3	37(2.27)
	Big rocks	14	14	19	13	17	77(4.72)
	Streams	8	5	2	2	1	18(1.10)
**(11.60%) Historic culture (11.60%)**	Cliffs	5	3	3	1	1	13(0.80)
	Ancient roads	2	1	0	2	3	8(0.49)
	Temples	12	9	8	6	3	38(2.33)
	Carved stones	9	4	1	11	2	27(1.66)
	Taoist temples	5	3	2	1	3	14(0.86)
	Statues	1	3	0	3	0	7(0.43)
	Relics	1	6	3	0	7	17(1.04)
	Door plaques	4	0	2	3	2	11(0.67)
	Memorial gates	2	4	5	7	2	20(1.23)
	Scriptures	3	0	0	2	0	5(0.31)
	Stone tablets	2	3	6	7	8	26(1.60)
	Courtyards	6	1	1	2	6	16(0.98)
**(5.40%) Native villages (5.40%)**	Farmhouses	7	13	8	12	13	53(3.25)
	Rape	0	0	2	0	0	2(0.12)
	Farmlands	3	2	4	3	2	14(0.86)
	Bamboos	1	0	0	2	4	7(0.43)
	Fruit trees	2	5	2	1	2	12(0.74)
**(25.46%) Mountaineering expedition (25.46%)**	Sticks	1	2	1	9	13	26(1.60)
	Backpackers	17	24	32	26	33	132(8.10)
	Advertising boards	1	1	1	4	1	8(0.49)
	Signage	11	11	11	23	20	76(4.66)
	Mountain roads	13	35	34	43	48	173(10.61)

**Fig 7:**
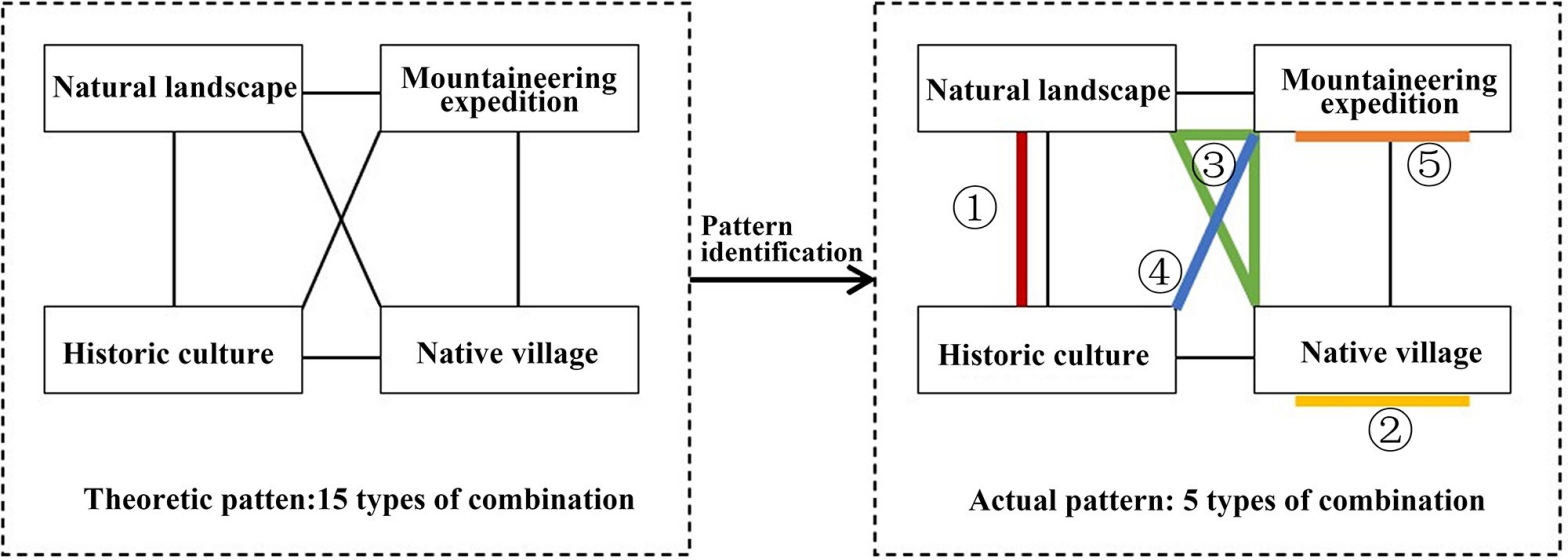
Theoretical and practical models of recreation preferences.

Theoretically, tourists visiting Ziwuyu have 15 recreation preferences, including four single and 11 mixed preferences. However, according to the actual results (Fig 7), there are five types of recreation preferences of tourists visiting Ziwuyu, of which two are single preferences, namely native villages and mountaineering expeditions, two are mixed preferences of two categories, namely natural landscape–historic culture and historic culture–mountaineering expedition, and one is a mixed preference of three categories, namely natural landscape–native village–mountaineering expedition. The recreation preference of each typical spatiotemporal behavior pattern is further explained here, and the probability of tourists visiting scenic spots was calculated through cross chi-square analysis to identify the risks of recreational behavior on the ecological environment of Ziwuyu.

Short-distance spatiotemporal behavior pattern: Tourists who exhibit this pattern have a strong leisurely nature. Tourists preferred "Natural landscape-historic Culture" for recreation, indicating that tourists in this pattern considered Ziwuyu a daily sightseeing and leisure destination. The probability of tourists in this pattern visiting Guaierya and Xiliang was 56.9% and 58.5%, respectively, and the probability of leaving the study area was 75.4%, as indicated by cross chi-square analysis. In addition, some tourists also visited Jinxian Temple, Xiaowutai, North Dongliang, and Yuandengtai, with a visiting probability of 41.5%, 26.2%, 13.8%, and 10.7%, respectively. In this pattern, tourists were concentrated in the north of the valley mouth, with less activity duration and small activity space, thereby having less impact on the environment.

Transversing spatiotemporal behavior pattern: The tourist recreation preference in this pattern was “native villages.” Cross chi-square analysis revealed that the probability of tourists visiting Qiliping in this pattern was 100%; thus, it was speculated that most of these tourists are local villagers, and Qiliping is the only way they have to pass during daily going out. After passing Tudiliang, the routes of many tourists started to disperse rapidly, with approximately 82.3% probability of leaving the study area by different routes. Among them, 50.6% of the tourists passed westward through Banliyakou to Cuibei Mountain to leave. In terms of the direction of travel, tourists in this pattern mostly passed through the valley mouth to arrive at Huangyu Temple, which is 1.3 km away from the west side of Banliyakou, or entered Fengyu.

Reentrant spatiotemporal behavior pattern: Tourists in this pattern exhibited the most comprehensive recreation preference, which can be summarized as “natural landscape-native villages-mountaineering expedition.” Cross chi-square analysis revealed that some tourists in this pattern did not turn back completely; instead, they visited Dayangjiao, Xiaoyangjiao, and Quanzhen Temple, with a visit probability of 33.3%, 32.2%, and 26.7%, respectively. Tourists in this pattern exhibited a 30% probability of visiting Jianshanding, a key protected area stipulated in the Regulations. Thus, Jianshanding may be faced with potential negative impacts from tourists.

Large-loop spatiotemporal behavior pattern: The recreation preference of tourists in this pattern was “historic culture-mountaineering expedition.” Cross chi-square analysis revealed that the probability of tourists visiting Yuandengtai and Xiaowutai in this pattern was 100%, which is consistent with the results of the photograph analysis. The probability of tourists visiting South and North Dongliang was 38.8%, indicating that the proportion of people passing down the mountain via these two routes is comparable. In addition, a few tourists exited the mountain from the east side of Dongliang. The probability of tourists visiting Jianshanding in this pattern was found to be up to 89.8%, indicating that this is the most dangerous spatiotemporal behavior pattern for Ziwuyu. Therefore, appropriate controls, and guidance measures should be implemented based on the behavioral characteristics and recreation preferences of tourists.

Small-loop spatiotemporal behavior pattern: The recreation preference of tourists in this pattern was the “mountaineering expedition.” Cross chi-square analysis revealed that the probability of tourists visiting and consuming in Shili farmhouse restaurant was 27%. The probability of visiting Guaierya twice was 52.4%, indicating that it is an important node. Some tourists did not go through Guaierya and directly left from the valley mouth on the east side of Dongliang.

## Conclusions and discussions

### Application of tourism digital footprint in tourist impact management

Tourism digital footprint has often been used to study the characteristics of tourist spatial flow at the macro scale of cities [51]; however, its role in tourist impact management in ecotourism destinations has been neglected [10]. Based on the digital footprints of tourists visiting Ziwuyu, in this study, the specific path was explored to differentiate tourists’ spatiotemporal behavior patterns and identify their preferences from the perspective of tourist impact management in ecotourism destinations; in addition, the reliability of using tourism digital footprint for studying tourist impact management in ecotourism destinations was verified. Tourist impact management in ecotourism destinations seeks a balance between minimizing impact on resources and the environment and optimizing recreation experience quality. Using tourism digital footprints to study tourists’ spatiotemporal behavior patterns and recreation preferences can lead to the development of more flexible tourist management policies for ecotourism destinations.

Tourism digital footprint can provide high accuracy across time, and large quantities of tourist behavior track data, which can be used for analyzing tourist spatiotemporal behavior patterns in ecotourism destinations. Through the cluster analysis of spatiotemporal elements such as tour duration, route length, and the number of scenic spots visited in this study, the tourist behaviors of those visiting Ziwuyu were structured into five typical behavior patterns: short distance, transversing, reentrant, large loop, and small loop. Compared with the traditional behavior observation method, activity log, and questionnaire method, the research results on tourist behavior patterns through tourism digital footprints are more objective. Compared with GPS track surveys, tourism digital footprint provides more accurate information by avoiding tourists deliberately changing their behavior when being tracked. Geotagged photographs constitute an important part of tourism digital footprint. The recreation preferences of tourists in ecotourism destinations can be identified by analyzing the photograph content. In this study, the recreation preferences of tourists in Ziwuyu were summarized into “natural landscape,” “historic culture,” “native village,” and “mountaineering expedition,” as well as 15 theoretical patterns of their various combinations. By describing the actual recreation preferences of tourists visiting Ziwuyu as the five typical behavior patterns, we successfully established a correlation between tourists’ behavior patterns and recreation preferences. This correlation is of great significance for tourist impact management in ecotourism destinations because it can guide in optimizing the recreation experience of tourists with different behavior patterns and provide guidance and control through the design of management strategies to minimize the impact of tourists on resources and the environment. In summary, it can provide technical support for improved management of ecotourism destinations.

### Study of tourist behavior and minimization of impact on resources and environment

Accurately describing behavior patterns and recreation preferences of tourists in ecotourism destinations enables identifying the risks faced by the key protected areas in advance to minimize the impact of recreational behaviors on resources and the environment [14]. With a visiting probability of 89.8% and 30%, respectively, some tourists in the large-loop and reentrant behavior patterns had visited Jianshanding, the key protected area in Ziwuyu. There were significant differences in the behavior tracks of tourists in the key protected area. They preferred "historic culture-mountaineering expedition" and "natural landscape-native village-mountaineering expedition" for recreation. Therefore, the key protected area is at risk of ecological destruction. The study discovered that 63 people visited Jianshanding, the key protected area of Ziwuyu I, out of the 348 samples used, with an overall visit probability of 18.10% and an average duration of stay of about 30 min per person. In terms of the intensity of tourist activities in different behavior patterns, some tourists visited key protected areas in both the large-loop and reentrant behavior patterns, with visit probabilities of 89.8% and 30%, respectively. There are clear differences in their behavior tracks within the key protected areas. Their preferred recreational activities are "historic culture—mountaineering expedition" and "natural landscape—native villages—mountaineering expedition" with "mountaineering expedition" being the most common. Tourist trampling may harm Jianshanding’s surface vegetation, wildlife habitat, water resources, and soil. Therefore, the key protected area faces the risk of ecological environmental damage. Although the tourists in the small-loop behavior pattern did not visit Jianshanding, their recreation preference was “mountaineering expedition,” which should also be focused upon.

In this regard, in the scenic planning of Ziwuyu, the road from Tudiliang to Cuibei Mountain shall be kept open, and the node design and service facilities in Cuibei Mountain shall be reinforced. As a general protected area with an altitude of 1471 m, Cuibei Mountain meets the basic conditions to replace Jianshanding to meet the mountaineering needs of tourists. Historic cultural landscapes must be constructed along the path from Tudiliang to Jianshanding to weaken the attributes of mountaineering, and signage must be installed at Tudiliang to divert tourists on mountaineering expeditions to Cuibei Mountain, and necessary control measures must be implemented. With the aid of digital footprint, we revealed the differentiation of spatiotemporal behavior patterns and preferences of tourists in ecotourism destinations to support the humanistic transformation of ecotourism destination management. In other words, tourists should be considered the subject of activity, transforming from the control of only tourists to finding alternative solutions in combination with environmental carrying capacity and tourists’ recreation preferences. Furthermore, appropriate guidance and control measures should be adopted through design strategies to achieve a balance between the sustainable development of ecotourism destinations and the recreation experience of tourists, which is also the core goal of tourist impact management [52]. We found that many tourists visiting Ziwuyu choose to leave the study area from the non-entrance, and they may have visited other key ecological reserves. Thus, the scope of research must be expanded in future studies, deeming the various valleys in the northern foothills of Qingling Mountains as a whole to identify the behavior patterns and recreation preferences of tourists between and in valleys and the negative effects they may bring to the key protected areas must be evaluated more accurately.

### Study of tourist behavior and maximization of recreation experience quality

The research results of behavior patterns and recreation preferences of tourists reported herein can guide tourist destinations to improve the design and layout of the facilities, which is of great significance to the optimization of tourists’ recreation experience in ecotourism destinations [45]. Through the cluster analysis performed in this study, the tourist behaviors of those visiting Ziwuyu were summarized into five typical behavior patterns. In addition, the recreation preference of tourists in each pattern was accurately identified through photograph analysis. In the transversing behavior pattern, tourists preferred native villages for recreation, and their probability of visiting Qiliping was 100%; however, their recreational activities can have a negative impact on the lives of local villagers. Therefore, tourist destinations should use the transversing tour route as a model for developing effective management strategies to guide and control tourism activities. The tourists’ recreational preference for traversing behavior pattern is "native villages" with the possibility of visiting Qiliping Village of 100%. Therefore, their recreational activities may have an impact on the spatial environment of local villagers, such as the potential negative impact of road congestion caused by tourists taking photos and the degradation of the quality of residents’ living environment caused by littering. It is recommended that the scenic spot use the traversing pattern’s travel path as a reference to develop effective management strategies to provide guidance or control. Tourists in the small-loop behavior pattern exhibited a 27% probability of visiting Shili farmhouse restaurant. Thus, dining facilities must be arranged according to the recreational characteristics of the small-loop pattern. In addition, more than half of the tourists in this pattern visited Guaierya twice, indicating that it is one of the important nodes. Thus, the construction of important nodes such as Guaierya must be emphasized, and corresponding service facilities must be established around them. Furthermore, we discovered that few tourists visit Quanzhen Temple, and both short-distance and large-loop behavior patterns have recreation preferences that include "historic culture." To improve the overall tourism value of Ziwuyu, corresponding design strategies must be adopted to enhance the spatial vitality of nodes such as Quanzhen Temple, and a "historic culture" tourism route must be created in combination with the recreational characteristics of tourists, running through Quanzhen Temple, Jinxian Temple, Xiaowutai, and Yuandengtai. Furthermore, we discovered that "historic and cultural" elements are present in tourist recreational preferences in short-distance and large-loop behavior patterns. It is suggested that the scenic spot combine tourist recreational characteristics to create a "historic cultural" Ziwuyu traveling route, connecting Jianshanding, Xiaowutai, Yuandengtai, and other tourist attractions characterized by religious culture, to increase Ziwuyu’s overall tourism value. The aforementioned findings demonstrate that tourist behavior research based on tourism digital footprint can aid in organizing and optimizing the spatial structure of ecotourism destinations, coordinating the behavioral conflict between tourists and local residents, guiding tour route design and tourism product supply, and serve as the basis for the collocation optimization of public service and commercial facilities [53], which are of great value for the maximization of tourists’ recreation experience.

## Research deficiencies and future research

For tourist impact management in ecotourism destinations, in this study, we proposed a method to differentiate spatiotemporal behavior patterns and recreation preferences of tourists and conducted empirical research by taking Ziwuyu as the research object and using the tourist tracks and photographs as the digital footprint. The research findings presented in this paper will serve as a reference to guide the refined management of ecotourism destinations. First, as a single data source, specific groups can be screened out using the digital footprint [17]. Furthermore, there is a scarcity of information on tourist attributes obtained from the Internet, limiting extensive research on causal relationship identification and tourist attribute differentiation. Future studies should use multisource data to accurately describe the behavioral characteristics of tourists in ecotourism destinations. Second, the number of tourists visiting ecotourism destinations during the holidays differs greatly from that during normal times; thus, different response plans for tourist impact management should be developed. In the short term, the ideas and methods proposed in this study are also applicable to identifying behavior patterns and recreation preferences. In the future, digital footprints could be used to study holiday tourist behavior in ecotourism destinations. In addition, the similarities and differences between the short-term and long-term characteristics of tourists’ recreational behaviors must be studied [46] to improve the management system of ecotourism destinations. Third, a recent study by Frey E et al. provided empirical evidence that tourists’ tour motivation may affect their spatial behaviors [54]. By determining the correlation between tour motivation and tourists’ behavior patterns and their recreation preferences, the management department of ecotourism destinations can adopt measures to provide tourists with opportunities to realize their tour motivation while reducing the ecological impact [16]. Tour motivation should be fully considered in future studies on recreational behaviors for tourist impact management. Fourth, we worked on this paper for three years, including 40 days during the COVID epidemic. The corresponding sample size is only 0.57%. It is difficult to compare the characteristics of tourist behavior with the epidemic as the time node. In the future, the potential impact of epidemic factors on tourist spatiotemporal behavior and preferences should be fully considered. Finally, some researchers have begun focusing on the study of tourist behavior prediction by using the Markov model [55], heuristic prediction algorithm (HPA) [56], long short-term memory (LSTM)-based deep learning model [57], and other methods to predict the mobility of tourists, which is of great value to tourist impact management. However, few such studies have been conducted on ecotourism destinations and should be the focus in the future.
